# Failing to retain a new generation of doctors: qualitative insights from a high-income country

**DOI:** 10.1186/s12913-018-2927-y

**Published:** 2018-02-27

**Authors:** Niamh Humphries, Sophie Crowe, Ruairí Brugha

**Affiliations:** 1 Division of Population Health Sciences, Royal College of Surgeons in Ireland (RCSI), 123 St Stephens Green, Dublin 2, Ireland; 2 Present Address: Research Department, Royal College of Physicians of Ireland (RCPI), Frederick House, 19 South Frederick Street, Dublin 2, Ireland

**Keywords:** Health workforce research, Health worker retention, Health worker migration, Generation X, Health system research, Qualitative methods, Ireland

## Abstract

**Background:**

The failure of high-income countries, such as Ireland, to achieve a self-sufficient medical workforce has global implications, particularly for low-income, source countries. In the past decade, Ireland has doubled the number of doctors it trains annually, but because of its failure to retain doctors, it remains heavily reliant on internationally trained doctors to staff its health system. To halve its dependence on internationally trained doctors by 2030, in line with World Health Organisation (WHO) recommendations, Ireland must become more adept at retaining doctors.

**Method:**

This paper presents findings from in-depth interviews conducted with 50 early career doctors between May and July 2015. The paper explores the generational component of Ireland’s failure to retain doctors and makes recommendations for retention policy and practice.

**Results:**

Interviews revealed that a new generation of doctors differ from previous generations in several distinct ways. Their early experiences of training and practice have been in an over-stretched, under-staffed health system and this shapes their decision to remain in Ireland, or to leave. Perhaps as a result of the distinct challenges they have faced in an austerity-constrained health system and their awareness of the working conditions available globally, they challenge the traditional view of medicine as a vocation that should be prioritised before family and other commitments. A new generation of doctors have career options that are also strongly shaped by globalisation and by the opportunities presented by emigration.

**Discussion:**

Understanding the medical workforce from a generational perspective requires that the health system address the issues of concern to a new generation of doctors, in terms of working conditions and training structures and also in terms of their desire for a more acceptable balance between work and life. This will be an important step towards future-proofing the medical workforce and is essential to achieving medical workforce self-sufficiency.

**Electronic supplementary material:**

The online version of this article (10.1186/s12913-018-2927-y) contains supplementary material, which is available to authorized users.

## Background

Medical workforce planning is about *‘getting the right staff with the right skills in the right place at the right time’* [1] to ensure that healthcare is delivered. The failure of high-income countries to achieve a sustainable medical workforce inevitably leads to a reliance on internationally trained doctors, often from low-income countries already experiencing doctor shortages [2, 3]. These countries struggle to deliver care [2], leaving health systems weakened, unable to achieve the health goals for sustainable development and ill-equipped to respond to disasters [4]. These challenges, which have been long-associated with low and middle-income countries (LMICs) [2], are becoming a feature of health systems in high-income countries, such as Greece and upper middle-income countries, such as Bosnia-Hercegovina [5, 6].

This paper focusses on a high-income country, Ireland, which has recently invested significantly in medical training [7], but which has not achieved medical workforce self-sufficiency. Ireland has doubled the number of doctors trained annually from 340 in 2007 to 725 Irish/EU doctors in 2016^1^ [8], but despite this, remains heavily reliant on internationally trained doctors to staff its health system, accounting for 36% of all registered doctors [9]. Ireland is one of the five OECD countries most heavily reliant upon internationally trained doctors [9], a dependence which has not reduced in recent years [10, 11], despite increases in the number of doctors trained.

The challenge to understanding doctor emigration in the Irish context is that the Irish health system does not systematically record doctor emigration, although a National Employment Record introduced by the Health Services Executive (HSE) in 2015, holds promise in this regard [12]. Ireland is not the only health system to generate limited or no data on the outward migration of health professionals [13]. In the absence of national data on doctor emigration, the best available indicator of emigration intent comes from verification data - 1881 verifications were issued to doctors practicing in Ireland during 2015 (Medical Council of Ireland data cited in [14]. As verification data is a measure of emigration intent rather than emigration [15, 16], the authors sought an alternative measure of doctor emigration via destination country immigration/registration data. This analysis revealed that between 2008 and 2014, 3798 doctors migrated from Ireland to five key destination countries - Australia, UK, Canada, New Zealand and the USA. This represents a significant outflow of doctors [14].

Ireland’s continued reliance on internationally trained doctors is out of sync with national and international policies promoting medical workforce self-sufficiency [4, 17], a fact recognised by Ireland’s national medical workforce planning agency [8]. The Irish health system is failing to retain enough doctors to staff the Irish health system, as indicated by an increased spend on agency staff from 2010 onwards [18]; the increasing reliance of hospitals on agency staff [7] and the emerging crisis in hospital consultant recruitment [19]. One reason for the persistence of these medical workforce challenges is that the policy response has focussed on training and recruitment, rather than on retention. While the increase in the number of doctors trained in Ireland in recent years has been impressive, the approach seems to mirror that of low to middle-income countries (LMIC) which *‘try to train more in the hopes we will keep more’* [20]. To halve its dependence on internationally trained doctors by 2030, in line with World Health Organisation (WHO) recommendations [4, 17], Ireland must become more adept at retaining doctors.

This paper explores the generational component of Ireland’s failure to retain doctors. This new generation of doctors completed their basic medical training in a digital, globalised era. Those who graduated in the last decade have also experienced first-hand the impact of austerity on the Irish health system, experiences which shape their decisions to remain and work in or leave Ireland. Doctors who graduated from medical school since 1990 (see Table 1 [21]) expect to change jobs several times over the course of their careers and no longer expect to remain with one employer for life [21]. In this regard, their experiences reflect wider generational changes towards high career mobility [22]. For a new generation of Irish-trained doctors, generational trends towards a greater level of career mobility, combine with a tradition of medical emigration [14] to create a cohort of doctors who routinely use international mobility to secure better working conditions, training and career progression opportunities [23].

**Table 1:** Generational profiles within the medical workforce [21]

Characteristic	Veterans	Baby Boomers	A new Generation (Gen X & Y)^a^
Year of birth	1922–45	1946–64	1965–2000
Attitudes to work	Respect the system. Work for security.	Married to medicine Works long hours. Loyalty to employer.	Being a doctor is only one aspect of identity. Resist long working hours. Expect to change jobs. Value work-life balance & flexibility.
Reward	A job well done	Money, title, recognition	Freedom and time Meaningful work

^a^For the purpose of this discussion, the authors have grouped together the categories of Generation X and Generation Y as a new generation of doctors, whose experienced contrast with those of the Baby Boomer generation

### A new generation, a new approach to medicine

Alongside formal clinical training (basic medical training and postgraduate specialist training), medical students and trainee specialists learn to think, speak and act like doctors [24, 25] as they undergo a process of professional socialisation [26] in order to *‘be initiated into the status of physician’* [27]. The presence of generational differences within the medical workforce have been discussed in online fora [28] and by researchers [21, 29, 30] and may pose a challenge to the traditional view of medicine and the role of the doctor [21, 28–30] and may also signal a change in the culture of medical migration [31]. The role of doctor has been shaped by the Baby Boomer generation (see Table 1) whose approach to medicine was characterised by long working hours and the assumption that ‘*the answer to every problem was to work harder, no matter what the sacrifice’* [28]. The resultant socialisation process has attempted to instil in doctors an acceptance of the demanding nature of medicine and an understanding that sacrifice is required to achieve career progression [30]. However, internationally, a new generation of doctors is challenging the following central tenets of the Baby Boomer generation’s approach to medicine:

- Medicine is your life – other interests should be secondary [32].
- Do not take on personal responsibilities which might interfere with hospital work [33].
- Demonstrate toughness by working long hours without showing fatigue [33].
- Work long hours and stay at work late to build your reputation [26].
- Cope well with heavy workloads because *‘the busier the job, the better the training’* [34].
- Resist handing over your patients to anyone [26].
- Time spent abroad may benefit your career prospects [35].

Professional cultures are policed and enforced by senior members of the profession who are the gatekeepers to training and career progression, who decide *‘what sort of candidate would be a good ‘fit’ for a position’* [36]. The difficulty is that their vision of medicine does not align with that held by a new generation of doctors, which has begun to reject the workaholic values of an earlier generation [21]. They do not want to be *‘married to medicine’* [29], but rather to pursue purposeful work in medicine alongside a rich personal life [37]. In a recent survey of 1749 hospital doctors, Hayes et al., found that trainee doctors (38%) were more likely than their consultant colleagues (24%) to show signs of burnout [38] and were less likely to feel that they had achieved an acceptable work-life balance. While 50% of consultants surveyed felt that they had a very good or excellent quality of life, only 30–33% of trainees felt the same way [38].

### Austerity, migration and retention

Ireland’s doctor shortage is not a shortage of individuals qualified to practice medicine, but rather an unwillingness of qualified doctors to practice medicine under the current conditions [39]. Doctors, trained in Ireland, are opting to emigrate rather than practice medicine in the Irish health system. Ireland has had a long tradition of medical migration; however a high level of return migration meant that doctor migration could be considered a positive for the Irish health system [40]. A recent report outlining challenges recruiting hospital consultants [19] may stem from the reluctance of a new generation of emigrant doctors to return to practice in Ireland. This is borne out by recent research, which indicates that Irish trained doctors are migrating to Australia for the longer term [41]. As native English speakers, holding skills and qualifications that are in high demand, Irish trained doctors are highly sought after in destination countries. Previous research by the authors has shown that the longer doctors remain abroad, the less likely they are to return to practice in Ireland [15, 42]. Given the challenges faced, even by high-income source countries [43] in encouraging the return of emigrant doctors, retention must be seen as the key challenge facing the Irish medical workforce [8].

Like many OECD (Organisation for Economic Co-operation and Development) countries, Ireland has struggled to sustain a health workforce in a funding constrained environment [44, 45]. Since the onset of economic recession in 2008/9, the Irish health system has *‘endured radical resource cuts’* [46]: staffing levels have fallen by 13%, the number of acute hospital beds has fallen by 13% and hospitals are operating at 93% capacity [47]. The impact on the medical workforce has been predictable and significant – doctors are dissatisfied with staff shortages, heavy workloads and long working hours [48]. They feel undervalued and are concerned about the quality of care that they provide [49]. They are unhappy with the reduced salary scales for new-entrant consultant  and the heavy workloads expected of them in the Irish health system [50, 51].

Rather than endure the difficult working conditions within an austerity-constrained health system [15, 42], Irish-trained doctors opt to emigrate. Although retaining doctors and reducing Ireland’s over-dependence on internationally trained doctors are national policy objectives [8], in line with recommendations from the World Health Organisation (WHO) [4, 17], Ireland continues to have a high rate of doctor emigration. This has created a recruitment and retention crisis even at consultant level [19]: of 149 consultant posts advertised in 2015, 20 received no applicants and 28 had only one applicant [19]. In 2016, one in five approved permanent consultant posts were either vacant or had been filled on a temporary basis [52]. This paper explores why Ireland is failing to retain a new generation of doctors and the impact that austerity, generational change and globalisation are having on doctor retention.

## Methods

The Doctor Emigration Project is a research project funded by the Irish Health Research Board (HRB). Research ethics approval for the study was obtained from the Royal College of Surgeons in Ireland Research Ethics Committee This paper presents findings from in-depth interviews conducted with 50 doctors between May and July 2015. The COREQ criteria for reporting qualitative research have been used as a template for reporting the qualitative research methods used [53].

Recruitment: In 2014, a year prior to the interviews, respondents completed a survey by the Irish Medical Council, agreed to participate in further data collection by the Doctor Emigration Project research team and provided their contact details to enable follow up contact (see [54] for details on the quantitative survey). 342 of these survey respondents to invite them to participate in the qualitative interviews [55]; and interviews were conducted with forty-five of these respondents. Recruiting respondents was more challenging than anticipated. For those who gave a reason for their non-participation in the interview process, a shortage of time and the challenging logistics of scheduling an interview around work commitments played a significant role. An additional five respondents were recruited via snowball sampling. Interviewing continued until data saturation was reached at which point it was considered that sufficient data had been generated. Care was taken to interview a broad range of doctors across migration status, specialty, gender, family status, age and grade. Details of respondent characteristics are presented in Table 2.

**Table 2:** Respondent characteristics

	N	%
Sex		
Female	29	58
Male	21	42
Age		
25–30	18	36
31–35	26	52
36–40	5	10
41–45	1	2
Location at time of interview		
In Ireland	39	78
Abroad	11	12
Migration Plans at time of interview		
Already Abroad	11	22
Definite plans to migrate	15	30
Intend to remain in Ireland (until training complete)	24	48

Interviews: Interviews were conducted between May and July 2015. Five interviews were conducted by NH (or by NH and SC together, to ensure a consistent approach to interviewing) and forty-five by SC alone. In line with the COREQ guidelines [53], the following reflexive details on the research team are included. NH is female researcher who holds Social Science qualifications up to PhD level. She had no prior relationship with any of the respondents. At the time of the interviews, she had 9 years prior experience in health worker migration research and a strong interest in the topic. SC is a female researcher who holds masters qualifications in social science research methods. At the time of interview she had 1 year of applied research experience. Interviews were scheduled around respondents’ availability, time zone constraints and personal preferences. They were conducted by Skype, by telephone, or in person. Those conducted in-person were conducted at a time and place that best suited the respondent, including in offices within the research institution, in offices on hospital sites and in their home environment (a buddy system was in place to ensure respondent and researcher safety during data collection).

Interviews lasted approximately 60 min. All were audio recorded and transcribed verbatim by a third party. Interviews were guided by theme sheets (see Additional files 1 and 2). Theme sheets were developed following consultation with key stakeholders; one for emigrants and another one (with minor differences) for non-emigrant doctors. The theme sheets also covered the main themes raised in the international literature and also drew on our previous experience of researching health worker migration [3, 56]. Interviews covered respondents’ experiences of working in Ireland, their decisions to emigrate or to stay and the impact of that decision. Interviews concluded with a reflection on respondents’ future plans.

Data storage and analysis: Interview transcripts were stored and coded using MaxQDA software. Interview transcripts were coded on a line by line basis by two researchers (NH and SC), moving from thematic coding to analytic coding [57]. To protect their anonymity, respondents are referred to by number. No other identifiable information is reported. Two previous papers drawing on qualitative research findings have been published [14, 55].

## Results

At the time of interview, 10 respondents were emigrants, 15 were planning on staying in Ireland. Of the remaining 25 respondents, 15 had immediate plans to emigrate and the remaining 10 planned to emigrate on completion of their postgraduate medical training. Of those already living abroad, half were living in Australia, once again highlighting the importance of Australia as key destination for Irish trained health workers [41, 42]. When considering drivers of doctor emigration, there was remarkable consistency between respondent doctors who were planning to leave and those who planned to remain in Ireland. Forty-two of 50 respondents highlighted the importance of migration for career progression. In this regard, there was remarkable consistency between those who intended to remain in Ireland and those who planned to leave, with almost all considering international experience to be beneficial to the medical career. This illustrates the strength of the professional culture of migration in the Irish context [31]. They were aged between 24 and 39, with an average age of 32, meaning that all could be categorised as part of a new generation of doctors (see Table 1). Several themes emerged from the interviews which highlight the key issues facing this new generation of doctors and the reasons that Ireland is failing to retain them.

### Becoming a doctor

Respondents highlighted the challenge facing doctors in both completing their training and in practicing medicine – the long working hours, the heavy workloads and high work intensity experienced. They highlighted the fact that different generations within the workforce had contrasting ideas of being a doctor and the level of sacrifice necessary to practice medicine in Ireland.

*‘Younger consultants wouldn’t share that attitude that you have to work 70 or 80 h a week and*. *.*. *you have to have trial by fire. I do think that attitude is changing but it could take another 20 years for that to be the majority’* (Respondent 34).

Respondents highlighted that medicine has changed in recent years and a new generation of doctors are charged with treating an ageing patient population with complex co-morbidities [45, 58]. This has increased the workload and the work intensity for hospital doctors as *‘more patients are being treated, more quickly in acute hospital services*. *.*. *with patients on the wards for a shorter period of time, the average level of patient acuity is*. *.*. *higher, requiring more intense care’* [44]. Respondents felt that medicine was more demanding, and patient expectations of treatment higher, than had been the case for previous generations of doctors, as this respondent explains:

*‘There’s this mentality of ‘look, I went through it and I’m fine so you should be okay’. But . . . there's a lot more of a demand in your job than there was 20, 30 years ago’* (Respondent 6).

They challenged the traditional view of medicine as a vocation that should be prioritised before family and other commitments. They felt that medicine was a career rather than a calling or vocation, but were aware that this was not a view shared by senior doctors (of a previous generation), as this respondent explains:

*‘another NCHD*^2^ *. . . was told at a feedback session that . . . one of the negatives about her . . . that she prioritised her children above her job and this wasn’t a good thing. . . it’s almost as if she had to pretend she didn’t have children’* (Respondent 1).

### An austerity-constrained health system

For the current generation of doctors, their early experiences of training and practice have been in an over-stretched, under-staffed health system which is under strain as a result of several austerity budgets [47, 59]. Front-line health workers in Ireland have seen this impact on their working lives; on their salaries, terms and conditions of service; as well as on staffing levels and on the standard of care they can deliver. In a recent HSE staff survey, only 12% of medical/dental staff surveyed felt that they had the equipment, support and resources required to do their job correctly [60]; and consultants and trainees have both expressed concern about the quality of care that they provide [49].

In the process of becoming a doctor in an austerity-constrained health system, respondents in this study reported being *‘prepared to be overwhelmed’* (Respondent 26), describing steep learning curves within their early career pathways:

*‘The responsibility quadrupled in the space of five days’* (Respondent 18).

They felt unprepared for these transitions. They also felt that there was insufficient consultant support available to them during these transitions, as these respondents explain:

*‘. . . straight from an intern year to do an ED* (Emergency Department) *job and you’re on your own there from midnight to 8 am. . . And there’s no senior support or you just don’t ring the consultant.’* (Respondent 4).

*‘Often you just get thrown into a situation and you just get on with it and you just do it and you just hope that nothing dramatic does go wrong. . . when there isn't anybody who ’s able to come and help you because everybody else is overstretched as well’* (Respondent 14).

Working within an overstretched, understaffed health system had required trainees to behave as independent doctors at an early stage in their training, even if they felt unprepared for this level of responsibility, as this respondent explains

*‘I would have felt a bit of pressure to try and do a lot of it on my own and just cope, get on with it . . . Because if you start complaining or you start asking for too much help, then you're an extra burden on somebody who is equally as stressed and busy as you are*’ (Respondent 21).

Whereas the medical team was traditionally a source of support for junior doctors, respondents felt obliged not to let the team down. Although they knew in advance of the long working hours expected of doctors in the Irish health system, many struggled with ‘*the reality of working 100-hour weeks’* (Respondent 14). The scale and variety of the workload assigned to them as trainee doctors was also challenging:

*‘I probably hadn’t expected it to be as busy as it is or for there to be so many mundane tasks or so many patients, such long waiting lists, such an unstructured over-run health service. . . I didn’t quite realise it would be as bad as . . . it is’* (Respondent 29).

However, despite the difficult working conditions, long working hours and heavy workloads, respondents remained positive about their chosen profession, as one explained: *‘the work conditions are horrendous*. *. . but I love what I do so I can’t really complain’* (Respondent 6).

## Discussion

### A new generation of doctors

As Bickel and Brown have highlighted, the challenges facing a new generation of doctors differ from those faced by previous generations [29]; and, as this paper illustrates, these challenges are exacerbated by working within an austerity-constrained health system. Respondent insights highlight some of the challenges faced by a new generation of doctors working in health systems under strain. They face challenging working conditions [15, 48], stress and overwork [61]; they feel demotivated [18] and undervalued [15]; and they desire a greater level of workplace support. They struggle with low morale [62] and many are experiencing burnout. Recent research by Hayes et al. [38, 63] highlights the generational differences between consultant and trainee doctors in the Irish health system in that only 14–17% of trainees felt that they had enough time for their family/personal life in comparison to 28% of consultants. This may be because more senior doctors have more control over their working hours, but may also relate to the different expectations of what constitutes ‘enough’ time for family and personal life. In common with others of their generation [22], a new generation of doctors want to achieve a better work-life balance (see Table 1 [21]). This poses a challenge for the Irish health system, which is still struggling to comply with the European Working Time Directive (EWTD)^3^ and to achieve a consultant delivered health service [64], policy objectives initially flagged in 2003/4 [64]. Ireland’s health system has been slow to adapt to the needs of a new generation of doctors who, as is the case internationally, have begun to question the *‘unlimited work commitment’* [21] traditionally associated with successful medical careers.

The desire of a new generation of doctors for change, combined with the continued impact of austerity on the health system and the availability of better working conditions in other countries, presents a triple challenge to the Irish health system. This paper shows that a new generation of doctors reject the terms and conditions associated with being a doctor in the Irish health system and are willing to emigrate to avoid them. Perhaps this willingness to emigrate has always been present in the Irish health system and the change has been more subtle – a greater reluctance by emigrant doctors to return to practice in the Irish health system. This poses a major challenge to the Irish health system.

Similar shifts have been reported internationally [21, 28–30]. If unresolved, it provides a recipe for continued, large-scale doctor emigration and a continued reliance on internationally trained doctors to make up the shortfall [3]. This research provides medical educators and workforce planners with the opportunity to better understand a new generation of doctors [21]; and to adjust Irish health workforce policy accordingly. This knowledge should be used to inform and enhance retention policy and practice so that Ireland’s investment in basic and specialist medical training [7] bears fruit in the form of a stable and motivated medical workforce and a reduced dependence on internationally trained doctors, in line with WHO recommendations [4, 17]. As the authors have previously highlighted, *‘if the Irish health system is to achieve a sustainable health workforce, then health professionals must be able to access good working conditions, training and career progression in the Irish health system. Emigration to achieve these basics must become a thing of the past’* [15]*.*

Understanding the medical workforce from a generational perspective requires that the health system address the issues of concern to early career doctors, not only by improving working conditions and training structures, but also by responding to the desire of a new generation of doctors for a more acceptable balance between work and life. This will be an important step towards future-proofing the medical workforce and is essential to achieving medical workforce self-sufficiency.

### Health system context and response

The crises facing the Irish medical workforce do not occur in a vacuum, but within a health system deemed by national and international commentators to be approaching *‘total systems failure’* [65, 66]. When contemplating health system change, key healthcare leaders have warned that the Irish health system requires a radical overhaul, rather than minor improvements and have advised Ireland not to approach that change *‘through the lens of the current system’* [52]. The same is true of the medical workforce, which is facing an unprecedented crisis through its failure to retain doctors [15, 41, 42] and its failure to attract consultants into posts within the Irish health system [19].

Emigration has meant that the recent increase in the number of doctors trained annually has made a minimal impact on the Irish medical workforce [7]. There is a need to acknowledge that the challenge faced is not an absolute shortage of doctors, but rather a shortage of doctors willing to work within the current conditions [39]. Resolving this shortage and achieving medical workforce self-sufficiency will require a more comprehensive policy, not limited to increasing the supply of doctors (via domestic production or via international recruitment), but by implementing effective strategies to improve doctor retention.

The key policy response to date to Ireland’s poor record of doctor retention, was the 2014 Strategic Review of Medical Training and Career Structures [67], which made a number of recommendations for enhancing medical training and career structures. In the most recently available update [68] some progress is reported; however, trainee doctors who were consulted as part of the process reported that the initiative had resulted in *‘little tangible change or impact on their day-to-day working lives and training experience’* [68]. This highlights the need for policy to consider doctor retention at a national, workplace and the level of the individual doctor in order to ensure that policy change delivers tangible improvements to the working conditions of doctors in the Irish context.

The response of some senior members of the profession to the difficulties and frustrations experienced by early career doctors has been disappointing. While some consultants have advocated on behalf of more junior colleagues, outlining the failures of the current system and the toll it extracts from frontline health staff [38, 69], others have berated the new generation of doctors for a perceived *‘obsession with their own work life balance’* [70]*.* It is here that the generational distinctions within the profession are most apparent and where the need for change is most obvious. This phenomenon is not exclusive to Ireland, literature from the UK and the USA highlights similar issues – the need for *‘the government and wider NHS need to truly respect and value junior doctors’* [71] and warns against the widespread use of phrases *‘like ‘it was much worse in my day’. Such phrases were felt to devalue legitimate concerns about their work’* [61]. As Smith notes, *‘it is not acceptable to blame the younger generation of physicians for being who they are. If the profession cannot inclusively define itself for all dedicated physicians, it will fail’* [21].

## Conclusion

In conclusion, Ireland’s failure to offer solutions to the significant challenges faced by a new generation of doctors in the Irish health system means that doctors will continue to emigrate in order to train and to work in more supportive work environments internationally [72], leaving Ireland with a problem of medical brain drain, a high dependence on internationally trained doctors and a highly transient medical workforce. These challenges are not unique to Ireland, but perhaps the triple challenge of unattractive working conditions, aggravated by economic austerity and globalisation/migration, have made them more visible in the Irish context. Now that Ireland is graduating sufficient doctors, it must decide whether their contribution will be to the Irish medical system or to the health systems of destination countries: *‘we can attract or repel them, and we must choose which we wish to do’* [29]*.*

## Additional files

Additional file 1: Theme Sheet for Emigrant Doctors: The interview guide used for interviews with emigrant doctors. (PDF 297 kb)

Additional file 2: Theme Sheet for non-Emigrant Doctors: The interview guide used for interviews with non-emigrant doctors. (PDF 219 kb)

## Abbreviations

ED: Emergency Department
EU: European Union
EWTD: European Working Time Directive
HRB: Health Research Board
LMIC: Low and middle-income countries
NCHD: Non-Consultant Hospital Doctors
OECD: Organisation for Economic Co-operation and Development
WHO: World Health Organisation

## Footnotes

1 Ireland trains Irish and EU students as well as a large number of non-EU medical students. The fees paid by these non-EU students are a valuable income source for Ireland’s medical schools, but these students are not intended for the Irish medical workforce and so are excluded from these figures.

2 NCHD or non-consultant hospital doctor is a junior doctor in the Irish health system. All respondents were on recognised training schemes at the time of the first survey in 2014.

3 The EWTD seeks to limit the working hours of hospital doctors to a maximum of 48 h per week.
