# Supplementary material for: Failing to retain a new generation of doctors: qualitative insights from a high-income country
Source: BMC Health Serv Res. 2018 Feb 27;18:144. doi: 10.1186/s12913-018-2927-y (PMC5830046; doi:10.1186/s12913-018-2927-y)
Supplement: Supplementary file 1 — Theme Sheet for Emigrant Doctors: The interview guide used for interviews with emigrant doctors. (PDF 297 kb) [file 12913_2018_2927_MOESM1_ESM.pdf]

Draft Theme Sheet Doctor Emigration Project [Emigrants]

Background Information to Record

Gender (record)	Nationality	Age	Marital Status	Children

- In what country did you study medicine and in what year did you graduate?
- Did you complete direct or graduate entry medicine?

Working in Ireland

- What was the last post you held in the Irish health system before you emigrated (grade, speciality, and stage of training)?
- Did you feel supported in that role? (What supports were available to you?)
- Overall, how would you describe your experience of working as a doctor in Ireland? How did it compare to your expectations of being a doctor?
- How would you describe your experience of postgraduate medical training in Ireland?
- When deciding your speciality, which was more important to you, to be a specialist or to be a specialist in Ireland?

Decision to Emigration

- Tell me about the decision to emigrate (what point in your career did you first consider it, when did you decide that you would definitely emigrate?)
- What factors/events led to the decision to emigrate?
- What was your primary motivation for emigration? (Career progression, to obtain training, to travel)? [Use Typology?]
- Could you tell me a little about the process of finding a job abroad? (Where find information? help from colleagues/friends?)
- When did you emigrate?

Destination Country

- Tell me about your decision to emigrate to [this country] (Why this country?)
- What factors influenced the decision to emigrate to [this country]?

Emigration Experience

- Can you talk about the transition from working as a doctor in Ireland to working as a doctor in [this country]? (what made it difficult/what made it easier)
- How would you compare Ireland and [this country] in terms of your
    - Working conditions
    - Training opportunities
    - Career pathway
- What difference has emigration made to your life? (Personal and Professional, has it matched your expectations)?

Connections/Networks

- Do you have much contact with other Irish-trained health professionals in [this country]? (If yes, what difference has this made to your career?)
- How easy or difficult has it been to progress your career in this country?
- Have immigration/visa issues been an impediment to your career progression at all?
- Do you maintain contact with former colleagues in Ireland? (What kind of ties do you have?)
- How do you think emigration will affect your medical career?
    - (What effect do you think it might have on your career <u>in Ireland</u>?)

Future Plans

- What are your future plans? (To remain, to return, emigrate elsewhere?)
- Would you consider returning to work as a doctor in Ireland (Why? /why not?)
    - If yes, when might you return?
- What would influence your decision to return?
- What are your impressions of the Irish health system since emigrating?
    - Do you think things have changed since you left?
    - Where do you get information from – media/colleagues/combination?
- What changes to the Irish health system might encourage the retention of doctors in the Irish health system?
- Have you any other comments about the emigration of doctors from Ireland?
