# Supplementary material for: Failing to retain a new generation of doctors: qualitative insights from a high-income country
Source: BMC Health Serv Res. 2018 Feb 27;18:144. doi: 10.1186/s12913-018-2927-y (PMC5830046; doi:10.1186/s12913-018-2927-y)
Supplement: Supplementary file 2 — Theme Sheet for non-Emigrant Doctors: The interview guide used for interviews with non-emigrant doctors. (PDF 219 kb) [file 12913_2018_2927_MOESM2_ESM.pdf]

Draft Theme Sheet Doctor Emigration Project [Stayers]

Background Information/Demographics

Gender (record)	Nationality	Age	Marital Status	Children

- In what country did you study medicine and in what year did you graduate?
- Did you complete direct or graduate entry medicine?

Working in Ireland
- What is your current post within the Irish health system (grade, speciality and stage of training)?
- Do you feel supported in that role? (What supports are available to you?)
- Overall, how would you describe your experience of working as a doctor in Ireland? How does it compare to your expectations of being a doctor?
- How would you describe your experience of postgraduate medical training in Ireland?
- When deciding your speciality, which was more important to you, to be a specialist or to be a specialist in Ireland?

Decision to Stay & Impact
- Tell me about your decision to stay in Ireland (what point in your career did you decide to stay?).
- What factors influenced that decision to stay (work factors? Training factors? Personal factors? Combination?)
- Can you think of any specific events that helped you to confirm the decision to stay?
- What were the advantages, from your point of view, to staying in Ireland? (Are there any disadvantages?)
- Has the emigration of colleagues/friends influenced your decision to stay?

Impact of Decision to Stay
- What difference has the decision to stay made to your life (personal, professional)?
- What prospects for career progression are available to you here? What training opportunities here?
- How would you compare Ireland and other countries in terms of
    - Working conditions
    - Training opportunities
    - Career pathways

Emigration of Colleagues/Friends
- Has the emigration of medical colleagues had any impact on your workload or on your working environment?
- How has the emigration of medical friends/colleagues changed your perspective on emigration?

Future Plans & Reflection
- What are your future plans? (short-term/ long-term) (To remain, to emigrate?)
- How would you describe your career prospects in Ireland?
- What changes to the Irish health system might encourage the retention of doctors in the Irish health system?
- What changes to the Irish health system might attract back emigrant doctors who have emigrated?
- Have you any other comments about the emigration of doctors from Ireland?
